# High expression of DDR1 is associated with the poor prognosis in Chinese patients with pancreatic ductal adenocarcinoma

**DOI:** 10.1186/s13046-015-0202-1

**Published:** 2015-08-22

**Authors:** Yanmiao Huo, Minwei Yang, Wei Liu, Jianyu Yang, Xueliang Fu, Dejun Liu, Jiao Li, Junfeng Zhang, Rong Hua, Yongwei Sun

**Affiliations:** Department of Biliary-Pancreatic Surgery, Ren Ji Hospital, School of Medicine, Shanghai Jiao Tong University, 1630 Dongfang Road, Shanghai, 200127 P.R. China

**Keywords:** DDR1, Pancreatic ductal adenocarcinoma, Prognosis

## Abstract

**Background:**

Discoidin domain receptors 1 (DDR1), a subtype of DDRs, has been reported as a critical modulator of cellular morphogenesis, differentiation, migration and invasion.

**Methods and results:**

In this study, we investigated the expression of DDR1 and its clinical association in Chinese patients with pancreatic ductal adenocarcinoma (PDAC). Across a cohort of 30 patients, we examined DDR1 expression in paired PDAC and corresponding adjacent non-tumor tissues by real-time quantitative PCR (RT-qPCR), or western blotting. DDR1 expression is significantly higher in PDAC, as compared to normal adjacent tissue, confirming results from the Oncomine databases. We validated DDR1 expression by immunohistochemistry across a non-overlapping cohort of 205 PDAC specimens. Kaplan-Meier survival curves indicate that increased expression of DDR1 is associated with a poor prognosis in PDAC patients (*P* = 0.013). Multivariate Cox regression analysis identified DDR1 expression, age, N classification and liver metastasis as independent prognostic factors in PDAC.

**Conclusions:**

This study demonstrated that DDR1 can well serve as a novel prognostic biomarker in PDAC.

## Introduction

Pancreatic ductal adenocarcinoma (PDAC) is a devastating disease and the fourth most common disease-related mortality worldwide [1, 2]. The 5-year survival is approximately 6 %, and only 20 % of patients present with resectable disease [3, 4]. Few PDAC cases (less than 10 %) are diagnosed at early stages [5], largely due to the absence of specific symptoms, and as such patients often present with advanced stage disease. Hence, the identification of novel prognostic indicators has become a major topic of interest to the PDAC research community at large.

The discoidin domain receptor 1 (DDR1) belongs to a subfamily of Receptor tyrosine kinases (RTKs) characterized by the presence of an extracellular discoidin homology domain that function to modulates cell proliferation and differentiation. DDRs are non-integrin collagen receptors composed of two types, DDR1 and DDR2, independently activated by receptor-specific collagens binding at the discoidin domain [6]. DDR1 can be alternatively spliced into five isoforms (DDR1a-e). Recently, studies have suggested DDR1 participates in several critical cell processes including: adhesion, migration, proliferation, and invasion [7–9]. Moreover, expression of DDR1 is known to be dysregulated in multiple human cancers such as lung, breast, hepatic, and ovary cancers, suggesting a previously unappreciated role of DDR1 in tumor formation and progression [10–13]. In PDACs, however, the role of DDR1 remains to be uncharacterized.

In this retrospective study, we examined the expression pattern of DDR1 at the mRNA and protein level and explored the relationship of DDR1 expression with clinicopathologic parameters, including overall survival. We found that the expression of DDR1 was associated with poor prognosis of Chinese PDAC patients.

## Materials and methods

### Ethics statement

This research was approved by the Ethics Committee of Ren Ji hospital, School of Medicine, Shanghai Jiao Tong University, and written informed consent was obtained from each patient involved in this study.

### Patients and tissue specimens

We retrospectively analyzed clinical and pathological characteristics of 205 patients who had resectable infiltrating PDAC and underwent surgical resection at the Biliary-Pancreatic Department of Ren Ji Hospital, School of Medicine, Shanghai Jiao Tong University, China, from January 2002 to June 2014 (see Table 1). Patients who had history of other solid tumors, received preoperative chemotherapy, radiotherapy or other anticancer therapies were excluded from this study. Standard pancreatectomy was undergone with lymph node dissection in patients who had no evidence of distant organ metastasis. Routine chemotherapy with gemcitabine had been given to all patients after operation, but no radiation treatment was done in any of the patients included in our study [14]. An additional 30 fresh frozen tissues of PDAC and corresponding adjacent non-tumor tissues (located more than 2 cm apart from the tumor tissue in each case) were also obtained from the same department [15]. The diagnosis was confirmed by two clinical pathologists.

**Table 1:** Association between DDR1 expression and clinicopathologic features in patients with PDAC

		DDR1 Expression		
Characteristics		High	Low	*P* value
	Total	(*n* = 126)	(*n* = 79)	(*χ*2 test)
Age (years)				
Mean (years)	65.19	66.82	62.69	0.374
< 65	107	65	42	0.826
≥ 65	98	61	37	
Gender				0.891
Male	117	74	43	
Female	88	52	36	
Tumor location				0.273
Head	139	89	50	
Body/tail	66	37	29	
Size				0.551
≤ 2 cm	27	18	9	
> 2 cm	178	108	70	
Tumor differentiation				0.054
Well	11	10	1	
Moderate/poor	194	116	78	
T classification				0.697
T1	11	8	3	
T2	31	18	13	
T3	125	73	52	
T4	38	20	18	
N classification				0.858
Absent	136	83	53	
Present	69	43	26	
AJCC stage				0.949
Stage I	38	20	18	
Stage II	135	73	62	
Stage III	21	10	11	
Stage IV	14	7	7	
Liver metastasis				0.560
Absent	191	118	73	
Present	14	8	6	
Vascular invasion				0.801
Absent	177	109	68	
Present	28	17	11	
Vital status				0.912
Dead	172	106	66	
Alive	33	20	13	

AJCC staging is according to the 7^th^ edition of the American Joint Committee on Cancer (AJCC) staging system [29]

### RNA extraction and real-time quantitative PCR (RT-qPCR)

Total RNA from primary tumor and adjacent non-tumor tissue samples was isolated with Trizol reagent (Takara, Japan), and reversely transcribed through PrimeScript RT-qPCR kit (Takara, Japan) according to the manufacturer’s instructions [16]. RT-qPCR was performed using a 7500 RT-qPCR system (Applied Biosystems, Inc. USA) with a 15-μl PCR mix containing 0.5 μl of cDNA, 7.5 μl of 2*SYBR Green master mix (Invitrogen, Carlsbad, California, USA), and 200 nM of the appropriate primers (Invitrogen, Carlsbad, California, USA). Primer sequences set for DDR1 detection were as follows: forward primer: 5’-GCGTCTGTCTGCGGGTAGAG-3’, reverse primer: 5’-ACGGCCTCAGATAAATACATTGTCT-3’. The relative levels of mRNA expression were calculated based on the difference between amplification of DDR1 and β-actin RNA (forward: 5’-ACTCGTCATACTCCTGCT-3’, reverse: 5’- GAAACTACCTTCAACTCC-3’) using the 2^-ΔΔct^ method [17]. To minimize technical (run-to-run) variation between the samples, all samples were analyze d in the same run for both target genes and reference genes. All experiments were performed three times with three technical replicates.

### Western blotting analysis

Western blotting was performed as previously described [18]. The DDR1 antibody was purchased from Proteintech Inc. and species-specific secondary antibody was purchased from Cell Signaling, Beverly, MA. Bound secondary antibodies were detected by Odyssey imaging system (LI-COR Biosciences, Lincoln, NE).

### Tissue microarray (TMA) construction

Tissue microarrays (TMA) were constructed using diameter of 1.5-mm cores including 205 cases of matched tumor and non-tumor tissues specimens. After screening and marking representative spots of tissues, the tissues were punched out and squeezed into the paraffin array blocks.

### Immunohistochemical (IHC) staining and scoring

Immunohistochemical staining was performed on a tissue microarray (TMA) containing 205 paired PDAC samples as previous described [3]. Scoring was calculated according to the sum of the percentage of positively stained tumor cells: 0–5 % scored 0; 6 %–35 % scored 1; 36 %–70 % scored 2; more than 70 % scored 3 and the staining intensity: no staining scored 0, weakly staining scored 1, moderately staining scored 2 and strongly staining scored 3, respectively. The final score was designated as low or high expression group using the percentage of cells staining positive multiplied by the staining intensity as follows: “-” for a score of 0–1, “+” for a score of 2–3, “++” for a score of 4–6 and “+++” for a score of > 6; low expression was defined as a total score < 4 and high expression with a total score ≥ 4. These scores were determined independently by two senior pathologists. The scoring by the pathologists was done in a blinded manner.

### Follow-up

The postoperative follow-up included clinical and laboratory examinations. OS, a measure of prognosis, was defined as the time from the date of surgery to the date of death or the last follow-up examination.

### Statistical analysis

All statistical analyses were performed using SPSS 19.0 (SPSS Inc., Chicago, IL, USA) and GraphPad Prism 5 (San Diego, CA) software. DDR1 mRNA in the tumor and adjacent non-tumor tissue samples were compared using a paired-samples *t* test. The *Chi-square* test and *Fisher’s exact* probability method was used to analyze the relationship between DDR1 expression and clinicopathological characteristics. Survival curves were evaluated using the Kaplan-Meier method, and differences between survival curves were tested by the log-rank test. Cox proportional hazards regression model was used to examine univariate and multivariate hazard ratios for the study variables. Only significantly different variables in univariate analysis including DDR1 expression level, Age, N classification, Liver metastasis were entered into the next multivariate analysis [19]. A two-sided *P*-value < 0.05 was considered statistically significant.

## Results

### DDR1 is transcriptionally upregulated in PDAC

**Fig. 1:**
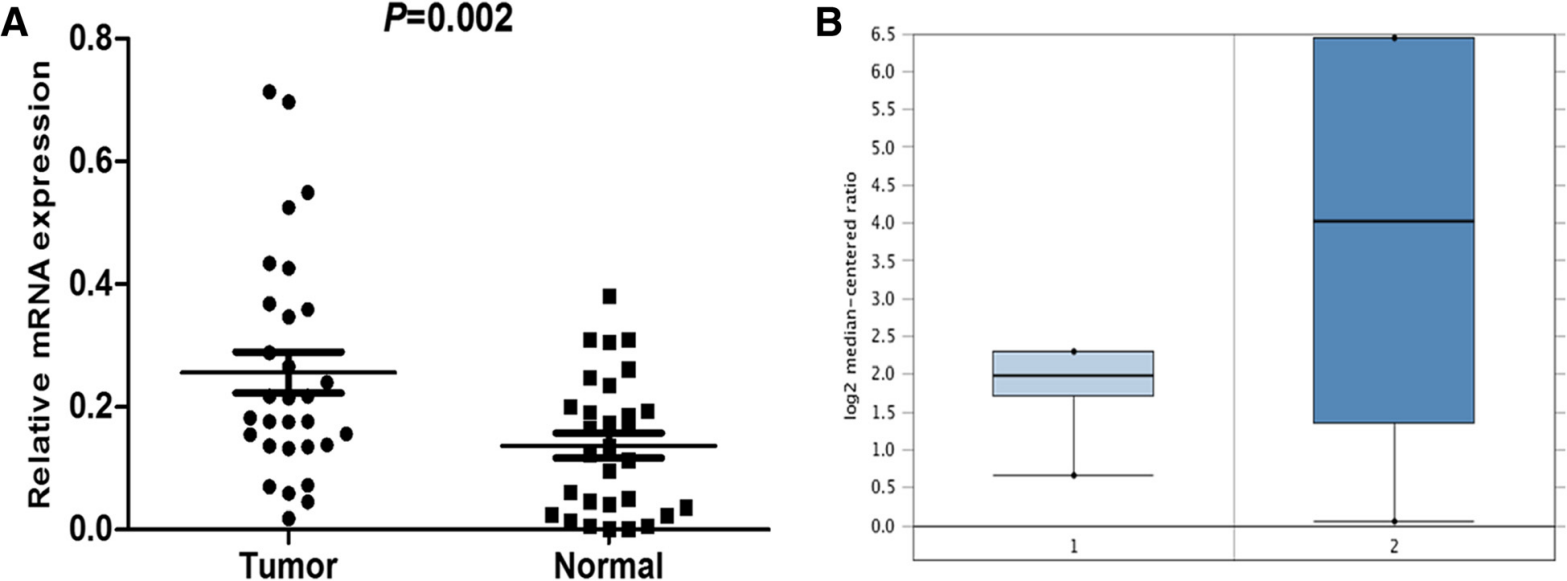
DDR1 expression is increased in PDAC at mRNA level. **a** increased DDR1 mRNA expression in 30 matched tumor (T) and non-tumor tissue (N) was detected by real-time quantitative PCR. **b** DDR1 expression in Buchholz pancreas grouped by normal pancreatic duct (1) and PDAC (2)

We evaluated the transcript levels of DDR1, by RT-qPCR, using 30 pairs of specimens from PDAC patients. The DDR1 mRNA levels were significantly increased in 19/30 PDAC tissue samples compared with the matched adjacent non-tumor tissue samples (*P* = 0.002, Fig. 1a). Additionally, we analyzed a microarray datasets from Oncomine databases showed in Fig. 1b [20] and found mRNA expression levels of DDR1 was consistent with our data.

### DDR1 expression at protein level in PDAC

**Fig. 2:**
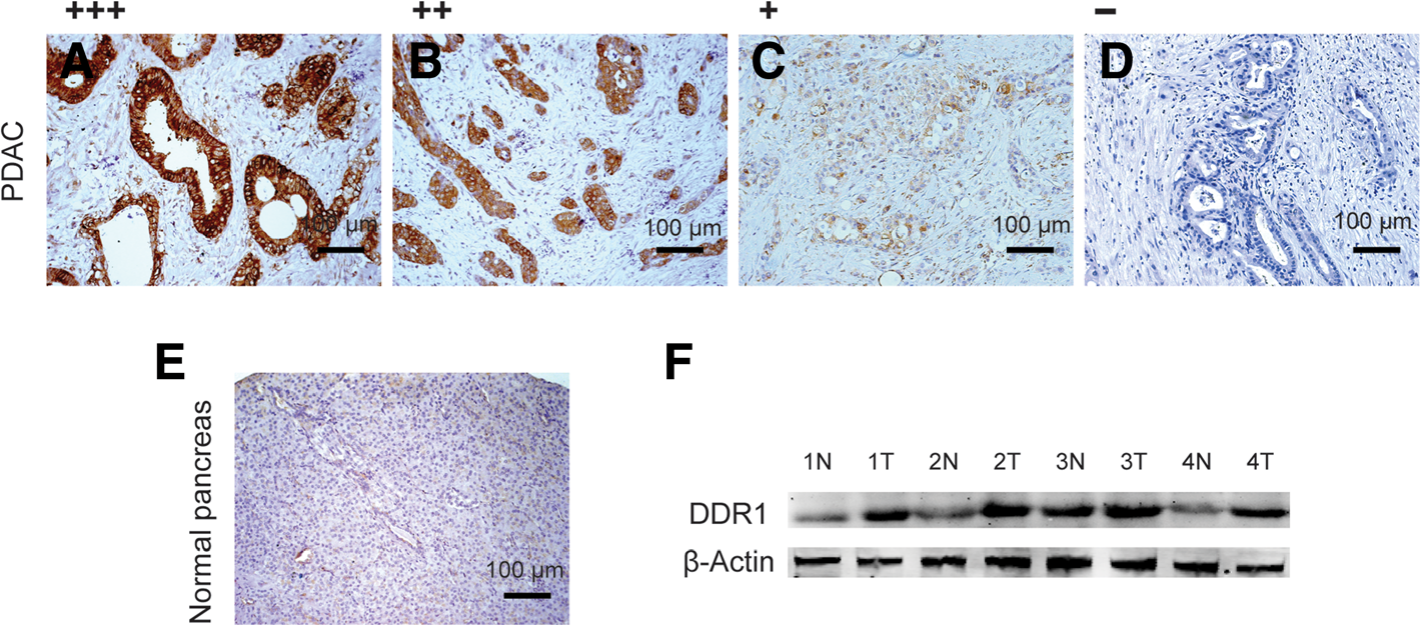
DDR1 protein expression in PDAC tissue samples. **a**-**e** Immunohistochemical representative images of DDR1 expression in PDAC compared with surrounding noncancerous pancreas (SNP) analyzed by immunohistochemisty; **a** PDAC, scored as (+++); **b** PDAC, scored as (++); **c** PDAC, scored as (+); **d** PDAC, scored as (-); **e** Normal pancreas, scored as (-). **f** Western blots of DDR1 expression in four pairs of PDAC patients

Based on the IHC scoring criterions established (see methods), 126 of 205 (61.5 %) PDACs specimens showed high DDR1 expression (DDR1 ++ or DDR1 +++), whereas the remaining 79 cases (38.5 %) displayed low DDR1 expression (DDR1- or DDR1 +) (Fig. 2 a-e). We valdated the protein overexpression across 4 pairs of resected representative specimens (tumor tissues and matched adjacent non-tumor tissues) from PDAC patients using Western blotting analysis. Consistent with the RT-qPCR results, an increase in DDR1 expression was observed in all of the 4 PDAC tissues compared with the matched adjacent non-tumor tissues (Fig. 2f).

### Correlation of DDR1 expression with clinicopathological features of PDAC

To determine the relationship between DDR1 expressions with the clinicopathological features of PDAC, the IHC staining of DDR1 levels were statistically evaluated by the *Chi-square* tests. The clinicopathologic parameters in PDAC included: age, gender, clinical stage, liver metastasis, vascular invasion and differentiation status. As shown in Table 1, no significant differences were found between DDR1 expression and any other parameters.

### Correlation of DDR1 expression and prognosis in PDAC patients

**Fig. 3:**
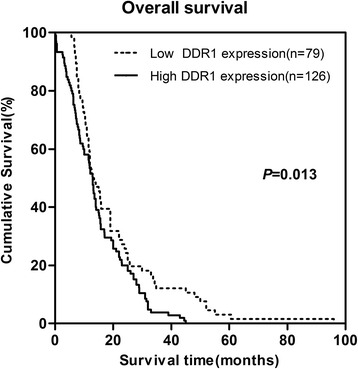
DDR1 expression is correlated with overall survival rate in PDAC patients. Kaplan-Meier survival curves show high expression level of DDR1 was significantly correlated with poor survival of PDAC (log-rank test: *P* = 0.009)

**Fig. 4:**
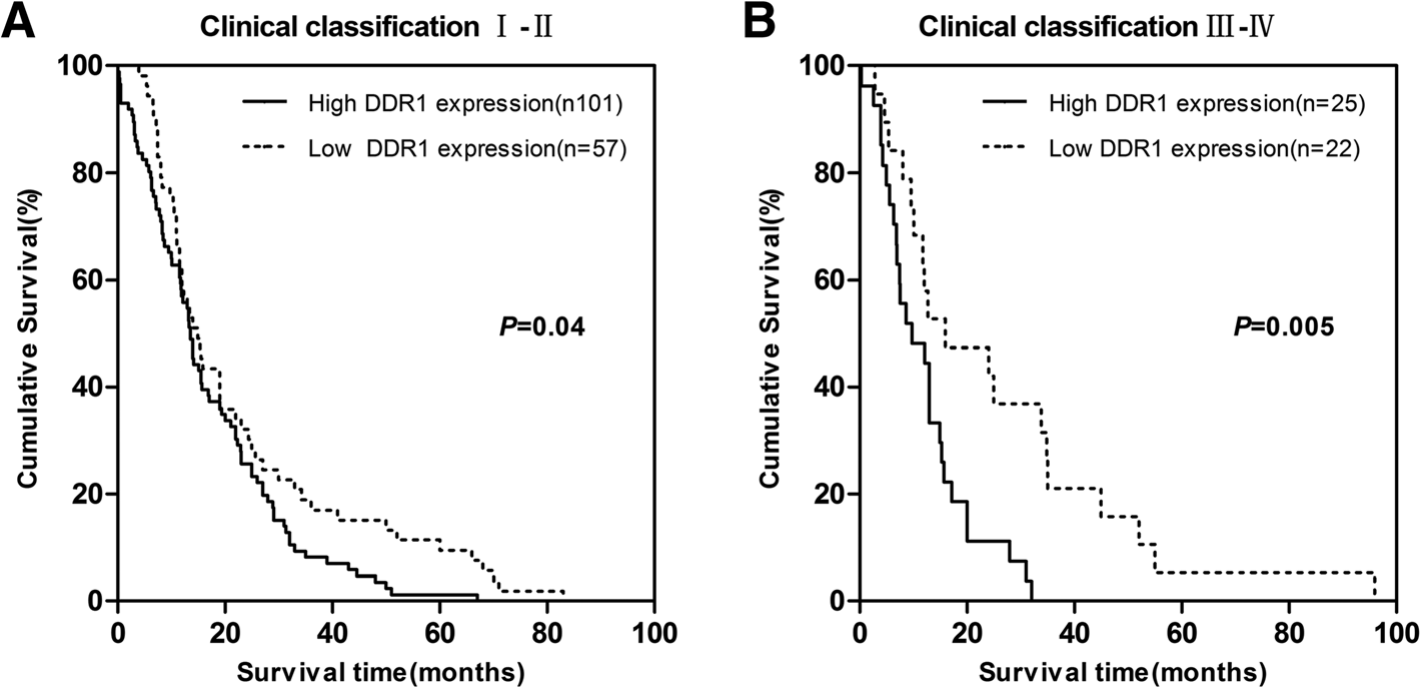
Comparisons of overall survival between DDR1 high expression and DDR1 low expression in early clinical stage (I-II) cohort and in advanced clinical stage (III-IV) cohort. *P*-values were calculated by log-rank test

**Fig. 5:**
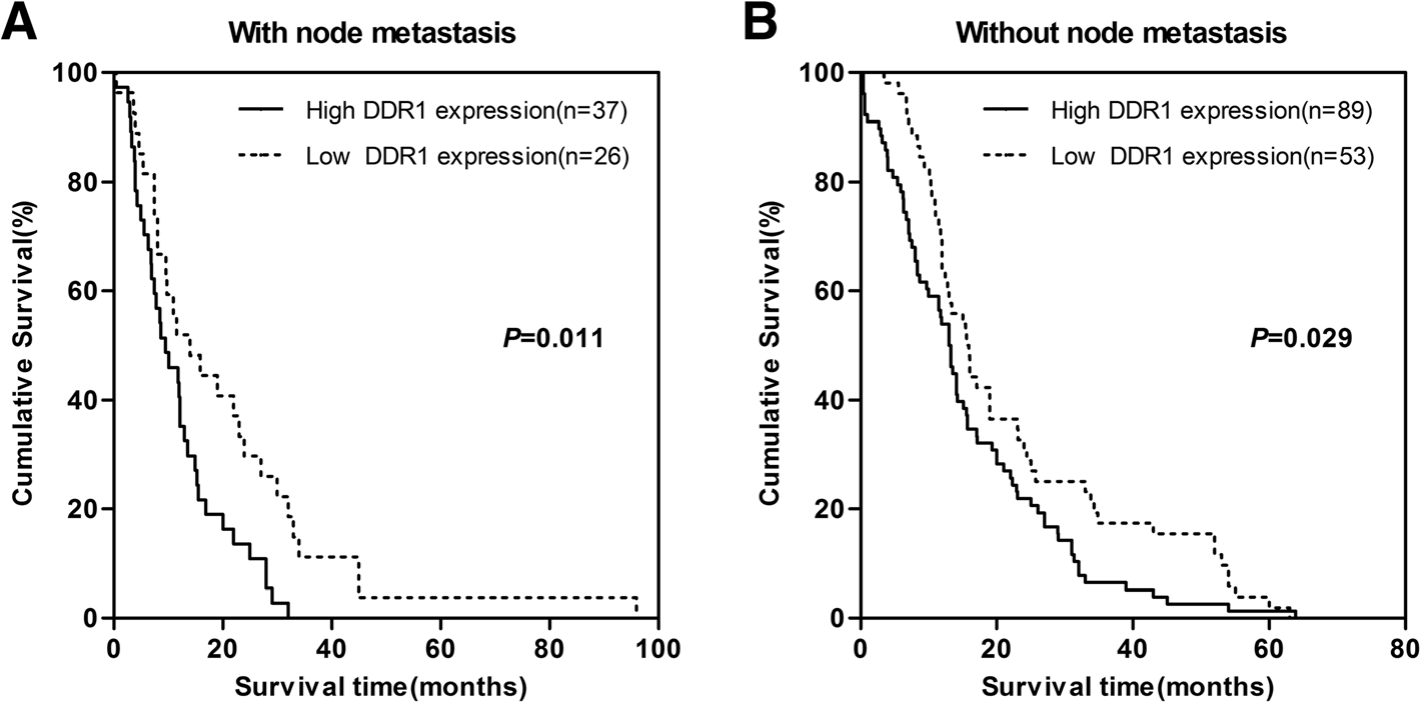
Comparisons of overall survival between DDR1 high expression and DDR1 low expression in patients with or without lymph node metastasis. *P*-values were calculated by log-rank test

**Fig. 6:**
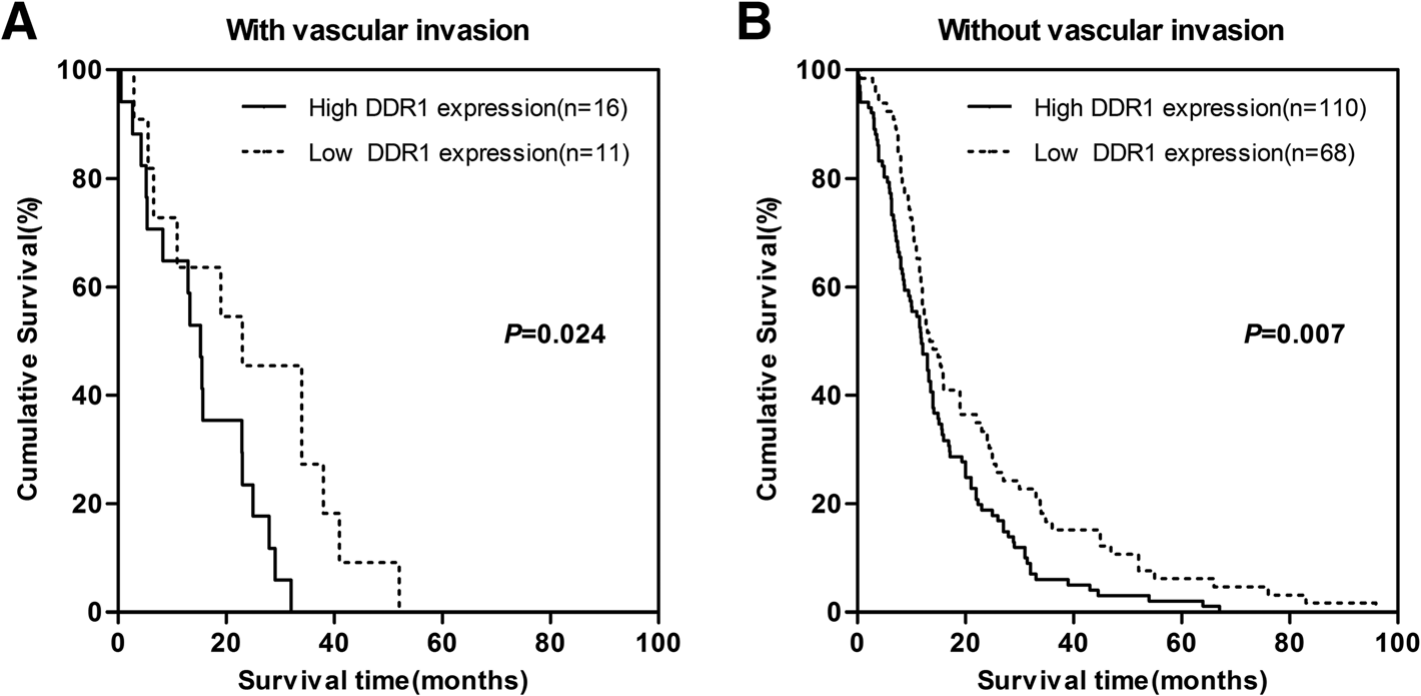
Comparisons of overall survival between DDR1 high expression and DDR1 low expression in patients with or without vascular invasion. *P*-values were calculated by log-rank test

The patients’ survival analysis was evaluated by Kaplan-Meier analysis and log-rank test. As shown in Fig. 3, there exists a negative correlation between DDR1 expression and overall survival (*P* =0.013). The association between DDR1 expression and overall survival in PDAC patients was also evaluated with regards to clinical stages, status of lymphatic metastasis and vascular invasion (Fig. 4, 5 and 6). The overall survival time was significantly different between patients with low and high DDR1 expression (*P* < 0.05), with the low DDR1 group having a longer overall survival independent of the clinical stage, status of lymphatic metastasis and vascular invasion.

### Univariate and multivariate analyses

Univariate and multivariate analyses were performed to compare the impact of DDR1 expression and other clinical and pathological parameters across our cohort (Table 2). Based on results of the univariate analyses, the age, DDR1 expression, N classification and Liver metastasis were significantly associated with overall survival. Furthermore, all these 4 factors were included in a multivariate Cox regression analysis to adjust for the effects of the covariates and were confirmed as independent prognostic factors.

**Table 2:** Univariate and multivariate analyses of prognostic parameters for survival in patients with pancreatic ductal adenocarcinoma (PDAC)

	Univariate analysis			Multivariate analysis		
Prognostic parameter	RR	95 % CI	P value	RR	95 % CI	*P* value
DDR1 (low vs.high)	0.613	0.439–0.857	0.004^a^	0.643	0.457–0.904	0.011^a^
Age (<65 vs. ≥65)	1.460	1.066–2.000	0.019^a^	1.533	1.107–2.122	0.010^a^
Gender (male vs. female)	0.765	0.552–1.058	0.105			
Tumor location (head vs. body/tail)	0.999	0.713–1.399	0.996			
Size (≤2 cm vs. >2 cm)	1.641	0.978–2.754	0.061			
Tumor differentiation (well vs. moderate/poor)	2.125	0.938–4.813	0.071			
T classification (T1 vs. T2 vs. T3 vs. T4)	1.104	0.871–1.400	0.412			
AJCC stage (I vs. II vs. III vs. IV)	1.260	0.992–1.600	0.059			
N classification (absent vs. present)	1.811	1.290–2.541	0.001^a^	1.769	1.242–2.519	0.002^a^
Liver metastasis (absent vs. present)	3.299	1.746–6.235	0.000^a^	2.748	1.419–5.320	0.003^a^
Vascular invasion (absent vs. present)	1.492	0.955–2.334	0.079			

*HR* hazard ratio, *CI* confidence interval

AJCC staging is according to the 7^th^ edition of the American Joint Committee on Cancer (AJCC) staging system [29]

^a^Statistical significant (*P* < 0.05)

## Discussion

Previously studies have identified DDR1 is overexpressed in various human invasive tumors including lung, breast, hepatic, and ovary cancers, highlighting its possible role in tumor initiation, maintenance or progression [10–13]. In the present study, DDR1 expression and its association with clinicopathological features including prognosis were investigated across a cohort of Chinese PDAC patients.

We determined that DDR1 expression was increased in Chinese PDAC patients at both the mRNA and protein level. These findings are supported by non-overlapping data from an Oncomine database, which highlights the same trends in PDAC. We expanded our analysis to a non-overlapping cohort of 205 patients and determined that DDR1 expression was upregulated in 126/205 PDAC specimens. Elevated DDR1 expression has also been reported in: (i) 52.2 % of hepatocellular carcinoma samples [11]; (ii) 61.0 % of non-small cell lung cancer [21]; and (iii) 63 % of serous ovarian cancer tissues [13]. The overexpression of DDR1 in these different human cancers support the hypothesis that DDR1 may impact tumorigenesis and/or tumor progression.

Previous studies indicated that DDR1 could promote tumor progression by inducing cell adhesion and differentiation, which might be due to: (i) coexpressing with adhesion molecules [22]; (ii) promoting epithelial-mesenchymal transition (EMT) [21, 23]; (iii) participating in functional interaction of Notch1 and NF-κB pathway [24, 25]. Furthermore, survival analysis in our study revealed that PDAC patients with high DDR1 expression levels had significantly shorter survival times than those with low expression levels. Univariate analyses showed that increased DDR1 expression was significantly associated with the overall survival rate in PDAC patients. Multivariate analysis demonstrated that DDR1 expression, together with some traditional prognostic factors, such as age, N stage and liver metastasis, were independent risk factors in the prognosis of PDAC patients. These results suggested that DDR1 may represent a novel prognostic marker for PDAC patients.

The precise molecular mechanisms through which DDR1’s impacts on tumor development and differentiation have yet to be elucidated. DDR1 presents 15 tyrosine residues in cell’s cytoplasmic regions, which are potential sites for phosphorylation and receptor activation by different types of collagens [6, 12, 26]. It has been shown that over-expression of DDR1 increased the migration and invasion of hepatoma cells *in vitro,* which implicated a role of DDR1 in tumor progression and metastatic dissemination [27]. Reduced or absent DDR1 expression *in vivo* leads to defects in placental implantation and development of mammary gland [28], while Miao et al.[21] demonstrated that DDR1 expression promoted epithelial-to-mesenchymal transition and contributed to non-small-cell lung cancer cells migration and invasion. The signaling pathways contributed by DDR1 upon cell-matrix interaction remain elusive and need further investigation.

In conclusion, this study demonstrated that DDR1 might serve as a novel prognostic biomarker in PDAC. Importantly, the molecular mechanisms underlying the relationship described above require clarification. Further studies are needed to investigate the molecular pathways involved in the regulation of DDR1, to improve our understanding and explore the possible therapies.
